# Automated vision system for fabric defect inspection using Gabor filters and PCNN

**DOI:** 10.1186/s40064-016-2452-6

**Published:** 2016-06-17

**Authors:** Yundong Li, Cheng Zhang

**Affiliations:** College of Electronics and Information Engineering, North China University of Technology, Beijing, 100041 China

**Keywords:** Machine vision, Fabric defect inspection, Gabor filters, PCNN

## Abstract

In this study, an embedded machine vision system using Gabor filters and Pulse Coupled Neural Network (PCNN) is developed to identify defects of warp-knitted fabrics automatically. The system consists of smart cameras and a Human Machine Interface (HMI) controller. A hybrid detection algorithm combing Gabor filters and PCNN is running on the SOC processor of the smart camera. First, Gabor filters are employed to enhance the contrast of images captured by a CMOS sensor. Second, defect areas are segmented by PCNN with adaptive parameter setting. Third, smart cameras will notice the controller to stop the warp-knitting machine once defects are found out. Experimental results demonstrate that the hybrid method is superior to Gabor and wavelet methods on detection accuracy. Actual operations in a textile factory verify the effectiveness of the inspection system.

## Background

Defect detection is highly important to fabric quality control. Traditionally, defects are detected by human eyes. The efficiency of this manual method is low and the missed rate is high because of eye fatigue. In the best case, a quality control person cannot detect more than 60–70 % of the present defects (Çelik et al. 2014b). Hence, an automatic inspection system is necessary for textile industry. In the literature, fabric defect detection methods were categorized into six groups: statistical, spectral, model based, learning, structural, and motif-based (Ngan et al. 2011). Spectral and structural methods, as well as defect classification by neural networks, are still popular topics in this field. Spectral methods include Fourier transform, wavelet transform, Gabor transform, and so on. The Fourier transform is the classic method for fabric analysis. However, Fourier transform was usually used with other approaches in the latest works (Schneider and Merh 2015; Hu et al. 2015; Mohamed et al. 2014; As et al. 2013). Schneider presented an automatic method for plain and twill fabric detection by combining Fourier analysis, template matching and fuzzy clustering (Schneider and Merh 2015). The system proved to be robust and versatile as a 97 % detection accuracy could be achieved. An unsupervised approach for the inspection of periodic pattern fabric by applying Fourier analysis and wavelet shrinkage was proposed (Hu et al. 2015). The advantage of this method is that no reference image is needed. Wavelet transforms elicited much attention in the fabric detection field because of its good local time–frequency characteristics (Zhu et al. 2015; Li et al. 2015; Hu et al. 2014; Wen et al. 2014). Wavelet methods perform well in defects with outstanding edges, but poorly in flat defects with smooth grayscale differences. Gabor filters are suitable for emulating the biological features of human eyes and were employed in fabric detection (Ibrahim et al. 2015; Hu 2015; Bissi et al. 2013; Jing et al. 2014). However, given that Gabor filters perform filtering of multi-scale and multi-orientation, which results in high computational complexity, real-time requirements are difficult to meet. To decrease the computational complexity, the optimal Gabor filter is built via genetic algorithm, in which the filter is only performed at one scale and one direction (Hu 2015; Jing et al. 2014). In recent years, neural networks have also been utilized for fabric defect detection and classification (Çelik et al. 2014a, b; Furferi and Governi 2008). Furferi and Governi proposed an ANN-based method to detect and classify defects, according to 23 parameters extracted from each acquired image (Furferi and Governi 2008). The advantage of this method is that no experimental thresholds are needed. In addition to the classic back-propagation (BP) networks, an emerging neural network, namely, Pulse Coupled Neural Network, is also applied to identify the defect area on plain fabric (Song et al. 2008; Zhu and Hao 2013).

**Fig. 1:**
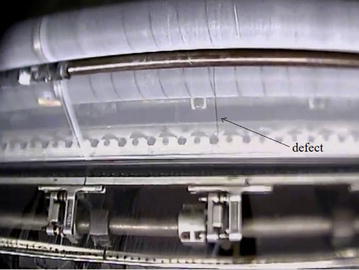
Common defects in warp-knitting fabrics

There may exist many types of defects in raw fabrics, such as loom fly, thin bar, broken end, etc. Furferi and Governi grouped these defects into three categories: dark and light area or point defects, dark and light localized defects and other defects (Furferi and Governi 2008). The most common defects of warp-knitting fabrics are linear defects of vertical orientation caused by the broken end of warp yarns (Du et al. 2012), which are shown as Fig. 1. The defects will become larger and larger if the warp-knitting machine is not stopped. So the target of an online vision inspection system is to detect defects and stop the warp-knitting machine as early as possible once defects appear on fabrics.

Though many researchers have focused on fabric defect detection in past years, it is still difficult to inspect defects of warp-knitted fabrics in practice due to the following reasons: (1) The quality of images captured by smart cameras in a factory is not as good as that in the laboratory because it is affected by lighting variation, machine vibration, dust, electromagnetic interference and other factors. Generally, the illumination device is very essential for image acquisition quality in machine vision system. However, to save cost and installation space, there are no specific lightening units in our system except fluorescent lamps installed along the web. (2) The typical defect caused by a broken yarn is not obvious, especially with the very thin yarn. All the above factors make defect detection is challenging in practice.

To deal with the difficulties, two issues are addressed in our study. First, Gabor filtering is performed on the images with specific parameters to enhance the image contrast. Second, a parameter adaptive PCNN is employed to segment the images with high precisions.

The other parts of this paper are structured as follows. The first section presents the system architecture. The second section proposes the fabric inspection algorithm. The third section focuses on the experimental results and discussions. The fourth section describes the actual operations. The final section concludes the research.

## System architecture

**Fig. 2:**
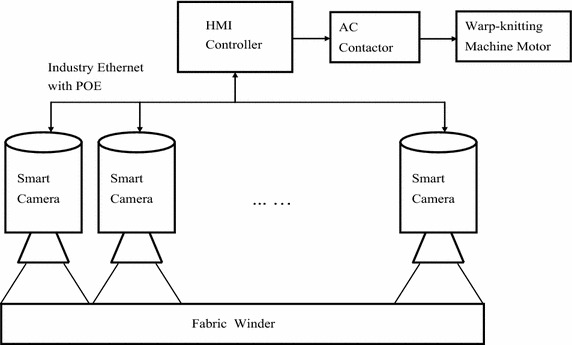
Automatic inspection system diagram

The inspection system consists of smart cameras and an HMI controller. Smart cameras are powered by POE, and can be accessed from the HMI controller through local area network. Alarm messages will be sent to HMI once a camera has detected a defect, and then HMI will trigger AC contactor to stop the main motor of warp-knitting machine. The system diagram is shown in Fig. 2.

In the past years, some machine vision systems have been developed to detect fabric defects automatically (Çelik et al. 2014b; Dorian et al. 2012, 2014). These systems have similar architectures, which consist of industrial cameras, frame grabber, lighting unit and a computer. In contrast to the existing PC-based methods, the advantages of the embedded system are obvious, which include small size, easy to install, low power consumption, low cost, etc. In practice, multiple cameras are installed on the beam of warp-knitting machine. Each smart camera covers about 900 mm width of the web.

**Fig. 3:**
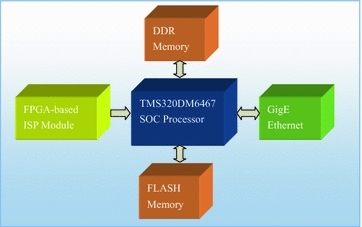
Hardware diagram of smart cameras

The smart camera consists of a SOC processor, FPGA-based Image Processing (ISP) module, DDR memories, FLASH memories and GigE Ethernet interface. Figure 3 shows the hardware diagram of the smart camera.

- ISP module: A low cost CMOS image sensor with 1920 × 1080 resolutions is employed, and then the raw data output from CMOS sensor are processed by an FPGA processor. After processing, image data are transferred into the memory of SOC in YUV422 format.
- SOC processor: TMS320DM6467 is chosen as host processor, which has an ARM9 core and a DSP core of 1 GHz. The detection algorithm is running on the DSP core and other tasks are implemented on the ARM9 core.
- GigE port: The Ethernet port is included for information interaction between smart cameras and HMI controller.
- Memory: There are128 MB DDR memories and 64 MB FLASH memories on the board.

## Hybrid method for fabric defect inspection

Fabric inspection algorithm is the key component of the smart camera software. The algorithm consists of two phases: image enhancement and image segmentation. Image enhancement is implemented by Gabor filtering with optimal parameters, which makes the defects more obvious. In this paper, a parameter adaptive PCNN is utilized to segment defects layer by layer.

### Gabor filters

A group of multi-scale and multi-orientation Gabor filters are suitable to characterize the texture features of the fabrics. So Gabor filters are widely used in the field of fabric defect inspection. The real part of the 2-D Gabor function is defined as:

1 $$g(x,y) = \exp \left\{ { - \frac{1}{2}\left[ {\left( {\frac{{x^{\prime} }}{{\sigma_{x} }}} \right)^{2} + \left( {\frac{{y^{\prime} }}{{\sigma_{y} }}} \right)^{2} } \right]} \right\}\cos (2\pi fx^{\prime} )$$

2 $$\left[ {\begin{array}{*{20}c} {x^{\prime} } \\ {y^{\prime} } \\ \end{array} } \right] = \left[ {\begin{array}{*{20}c} {\cos \theta } & { - \sin \theta } \\ {\sin \theta } & {\cos \theta } \\ \end{array} } \right]\left[ {\begin{array}{*{20}c} x \\ y \\ \end{array} } \right]$$

where *f* is the sinusoidal wave frequency, $\theta$ is the rotated orientation, $\sigma_{x}$ and $\sigma_{y}$ are variances along the *x*-axis and *y*-axis respectively.

**Fig. 4:**
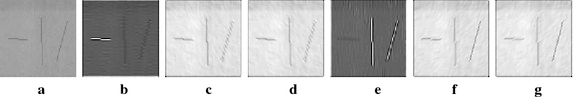
Results of Gabor filtering at different orientations. **a** The raw fabric image, **b** Gabor filtering with $0^{ \circ }$, **c** Gabor filtering with $30^{ \circ }$, **d** Gabor filtering with $60^{ \circ }$, **e** Gabor filtering with $90^{ \circ }$, **f** Gabor filtering with $120^{ \circ }$, **g** Gabor filtering with $150^{ \circ }$

The traditional Gabor filters perform filtering at multi-scale and multi-orientation, which result in high computational complexity. The real-time requirements are difficult to meet with our system. To simplify the Gabor filter operation, we only perform filtering at a specific orientation and scale. In fact, the outputs of Gabor filters are greatly affected by the parameter $\theta$ when applying Gabor filters to warp-knitted fabrics. Figure 4 gives a group results corresponding to different orientations. Figure 4a is the raw fabric image. The orientations of (b), (c), (d), (e), (f) and (g) are $0^{ \circ }$, $30^{ \circ }$, $60^{ \circ }$, $90^{ \circ }$, $120^{ \circ }$, $150^{ \circ }$ respectively. To demonstrate the effect of Gabor filter, three “handcrafted defects”, i.e., horizontal defect, vertical defect and diagonal defect, were added into the raw fabric image manually. It is clearly seen that the vertical texture defect is enhanced significantly by Gabor filtering with $90^{ \circ }$ orientation, which is shown as Fig. 4e. In contrast, the horizontal defect is enhanced by Gabor filtering with $0^{ \circ }$ orientation and greatly suppressed by Gabor filtering with $90^{ \circ }$ orientation. Since the defects of warp-knitting fabrics are usually of the vertical linear shape, so the Gabor filtering is only performed at the $90^{ \circ }$ orientation in our scheme.

We also found that the scale parameter doesn’t have much affection on texture features of warp-knitted fabrics. So only one scale is used in our method. In contrast to multi-scale and multi-orientation methods, our scheme can decrease the computation complexity, meanwhile remain the advantage of Gabor filters.

### Pulse coupled neural network

**Fig. 5:**
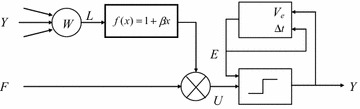
A simplified PCNN model

PCNN model, inspired by synchronous dynamics of neuronal activity in cat visual cortex, was developed by Johnson et al. on the basis of Echorn’s cortical model (Eckhorn et al. 1990; Johnson and Padgett 1999). Nowadays PCNN becomes the most potential method in image processing (Chen et al. 2011). In this study, we use a simplified version of PCNN (Zhu and Hao 2013) to decrease the computational complexity while remaining the advantages of cortical model. The simplified PCNN model is shown in Fig. 5.

PCNN is 2-D networks with a single layer. Each neuron of PCNN is corresponding to a pixel when applying PCNN to image processing. Suppose the image to be processed is *S*_*ij*_, *n* is the iteration number, PCNN in Fig. 4 can be described as follows:

3 $$F_{ij} (n) = S_{ij} (n)$$

4 $$L_{ij} (n) = \sum\limits_{k,l} {w_{ijkl} } Y_{i + k,j + l} (n - 1)$$

5 $$U_{ij} (n) = F_{ij} (n)(1 + \beta L_{ij} (n))$$

6 $$E_{ij} (n) = E_{ij} (n) - \Delta t + V_{e} Y_{ij} (n - 1)$$

7 $$Y_{ij} = \left\{ {\begin{array}{*{20}l} {1,} \hfill &\quad {U_{ij} (n) \ge E_{ij} (n)} \hfill \\ {0,} \hfill &\quad {otherwise} \hfill \\ \end{array} } \right.$$

where *F*(*n*) is the feeding input, *W* is the link weights which represents the impact of the around pixels, *Y*(*n*) is the binary state of each neuron, *L* is the linking input which is a convolution of *W* and *Y*(*n*). The internal activity term *U*(*n*) is the image pixel value modulated by the linking input. *E*(*n*) is the dynamic threshold of neurons. Once the internal activity *U*(*n*) is larger than the *E*(*n*), the neurons will fire and the sates in *Y*(*n*) will update to ‘1’. After firing, the dynamic threshold *E*(*n*) will increase suddenly, which makes the neurons couldn’t fire in a period.

There are four parameters in the simplified PCNN model: *W*, $\beta$, $\Delta t$ and *V*_*e*_. *W* is the link weights which represent the impact of the around pixels. Usually *W* is defined as the inverse of Euclidean distance. $\beta$ at the linking strength in the linking input. A larger value of *β* means a neuron is affected strongly by its neighboring neurons. $\Delta t$ is the decay step inverse to segmentation precision. A small value of $\Delta t$ could get a better segmentation precision. *V*_*e*_ is the amplitude of dynamic threshold *E*(*n*). Parameter setting is crucial to PCNN. Song proposed a learning method to determine optimal parameters from defectiveless reference images (Song et al. 2008). Chen et al. attempted to build a relationship between dynamic behaviors of neurons and the static properties of the image, and proposed an automatic parameter setting method based the relationship (Chen et al. 2011). Zhou proposed an automatic setting method based on the relationship between the dynamic threshold and the average of the firing area (Zhou et al. 2014). Herein, we present an adaptive setting method described as follows:

8 $$V_{e} = \hbox{max} (S_{ij} )$$

9 $$V_{aver} = \frac{{\sum\nolimits_{i = 1}^{m} {\sum\nolimits_{j = 1}^{n} {S_{ij} } } }}{m \times n}$$

10 $$\Delta t = \frac{{V_{e} - V_{aver} }}{N}$$

where *N* is the total iteration number. In addition to these parameters, the iteration number *N*_*best*_ with optimal segmentation should also be considered when using PCNN in defect detection. Researchers have developed some criterions to determine the optimal iteration number, such as Maximum Entropy, Minimum Cross Entropy, Maximum Variance of Inter-class and Minimum Variance of Intra-class. However, these criterions couldn’t obtain satisfied segmentation results. We propose a novel criterion based on firing time sequences, which is successfully used in defect detection of warp-knitted fabrics. The firing time sequences *T* (*n*) is defined as the number of fired neurons of iterations. The best iteration number is determined when *T*(*n*) increases suddenly, and the criterion is defined as:

11 $$N_{best} = n,\quad T(n + 1) - T(n) \ge 2T(n)$$

The defects can be segmented out from the fabric images accurately by PCNN iterations. After iterations we can get *N* frames of intermediate binary images. At last, the *N*_*best*_-th frame image is chosen as the final segmentation result. Suppose *S* is the input image, the iteration procedures are described as follows:

**Table:** 

Initialize *Y*(0), *E*(0), *W*, *β*, Δ*t* and *V*_*e*_
**for** *i* = 1 to *N* **do**
*F*(*i*) ← *S* *L*(*i*) ← *W* * *Y*(*i* − 1) *U*(*i*) ← *F*(*i*)(1 + βL(i)) *Y*(*i*) ← *U*(*i*) ≥ *E*(*i* − 1) *E*(*i*) ← *E*(*i* − 1) − ∆*t* + *V*_*e*_ *Y*(*i*) T(i) ← sum(*Y*(*i*))
**end for**
Determine *N*_*best*_ according to formula (11)

### Hybrid inspection method

**Fig. 6:**
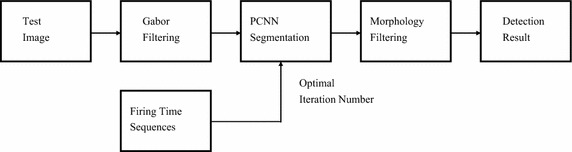
Flowchart of hybrid detection algorithm

In this section, a hybrid detection method combing Gabor filters and PCNN is presented. The flowchart of the method is shown in Fig. 6.

First, images captured by smart cameras are enhanced by Gabor filtering with $90^{ \circ }$ orientation. Second, defect areas are segmented by PCNN with adaptive parameter setting. Finally the optimal segmentation is determined according to firing time sequences, and noises are removed by morphology filtering.

## Experimental results and discusses

To evaluate the performance of the proposed hybrid method, we compared it with Gabor and wavelet methods. The testing code was implemented under MATLAB version R2012B. The testing computer was configured with an AMD Athlon processor with 3.01 GHz frequency and 3.25 GB memories.

**Fig. 7:**
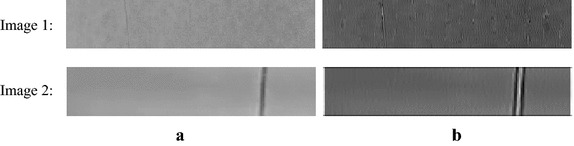
The effect of Gabor filtering. **a** Test images, **b** results of Gabor filtering

The available detection area of web on the warp-knitting machine is limited to a narrow rectangle. In the experiment, two images (labeled as Image 1 and 2) captured by smart cameras are used as test pictures, which are shown as Fig. 7a. The vertical linear defect is very unsharp. As mentioned before, the defect area is enhanced by Gabor filtering at specific orientation and scale. In this experiment, the parameters of Gabor filter are set as follows: $\sigma_{x} = 1.0,\sigma_{y} = 1.7$, $\theta = \pi /2,f = 5.5$. The filtering results of Gabor are shown as Fig. 7b. From Fig. 7, we can see that the defects are really enhanced.

**Fig. 8:**
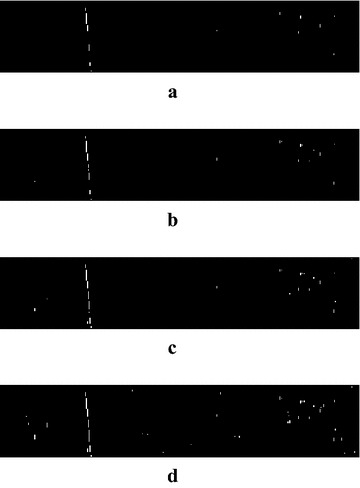
Intermediate segmentations of Image 1 by PCNN. **a** Result of the 10-th iteration, **b** result of the 11-th iteration, **c** result of the 12-th iteration, **d** result of the 13-th iteration

Next, the processed image is segmented by PCNN. $V_{e}$ and $\Delta t$ are determined according to (8)–(10). The total iteration number *N* = 20, $$W = \left[ {\begin{array}{*{20}c} {0.5} & 1 & {0.5} \\ 1 & 0 & 1 \\ {0.5} & 1 & {0.5} \\ \end{array} } \right]$$, $\beta = 0.1$. The image in Fig. 7b is segmented layer by layer, so we can obtain 20 frames of binary sequences. Figure 8 shows part of the intermediate segmentation results of Image 1.

**Fig. 9:**
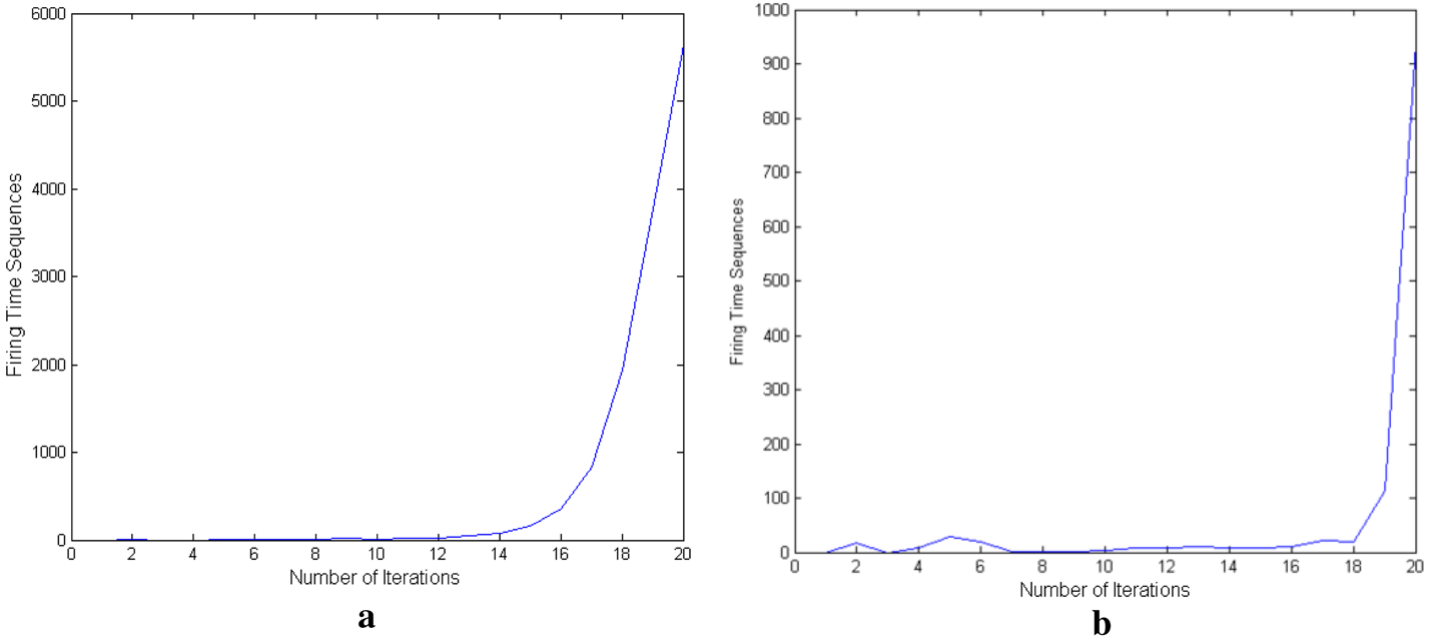
Firing time sequences. **a** Results of Image 1, **b** results of Image 2

How to choose the best result among the 20 iterations? Herein we propose an optimal criterion based on the firing time sequences of PCNN. Firing time sequences are the numbers of fired neurons of iterations, which include the temporal information of the image segmentation. The firing time sequence of Image 1 is shown as Fig. 9a. We can determine the best iteration number of Image 1 as 11 according to the formula (11). Figure 8 shows the segmentation results of the 10-th, 11-th, 12-th and 13-th iterations respectively. We can see that the 10-th segmentation is incomplete, while the 12-th and 13-th results include too many noises. So the 11-th segmentation is the best result. The firing time sequence of Image 2 is shown as Fig. 9b, and the best iteration number is determined as 17. However the intermediate binary results of Image 2 are omitted here to save space.

**Fig. 10:**
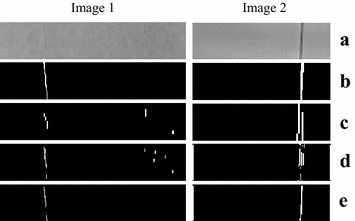
Detection results comparisons of wavelet (wavelet + binary thresholding + morphology filtering), Gabor (Gabor + binary thresholding + morphology filtering) and the hybrid (Gabor + PCNN + morphology filtering) methods. **a** The original images, **b** the ground-truths labeled by manual, **c** segmentation results of wavelet method, **d** segmentation results of Gabor method, **e** segmentation results of hybrid method

Figure 10 is the comparisons between the wavelet method, Gabor method and the proposed hybrid method. DB4 wavelets are employed to perform 2-level decomposition of the fabric images. Because the broken end defects are vertical linear, so the vertical sub-image at composition level 2 is used for defect detection. Figure 10c are the binary thresholding and morphology filtering results of the wavelet sub-image. To demonstrate the significance of the proposed hybrid method, Gabor only method is used for comparisons. Figure 10d are the binary thresholding and morphology filtering results of the Gabor filtering image. Figure 10e are the binary images segmented by the proposed hybrid method. Comparing to the ground-truths of Fig. 10b, we can see that the detection results of hybrid method are more accurate than other methods.

**Fig. 11:**
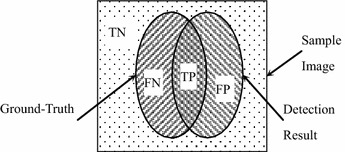
Definitions of TN, FN, TP and FP

Further, a group of metrics, including accuracy (ACC), true positive rate (TPR) and positive predictive value (PPV), is employed to quantify the detection accuracy (Li et al. 2016). The definition of ACC, TPR, PPV are described as (12)–(14)

12 $$ACC = (TP + TN)/(TP + TN + FP + FN)$$

13 $$TPR = TP/(TP + FN)$$

14 $$PPV = TP/(TP + FP)$$

where true positive (TP), true negative (TN), false positive (FP), false negative (FN) are labeled in Fig. 11.

The ACC, TPR and PPV metrics of the three methods are listed as Table 1, and the best results are marked in italics. We can see that the hybrid method is significantly better than the wavelet and Gabor methods, and get the highest scores of all testing items.

**Table 1:** Detection accuracy comparison of three methods

Test images	Methods	ACC	TPR	PPV
Image 1	Wavelet	99.23	15.61	21.09
	Gabor	99.37	42.20	41.48
	Hybrid	*99.70*	*44.51*	*98.72*
Image 2	Wavelet	96.68	23.42	18.57
	Gabor	96.58	23.42	17.81
	Hybrid	*97.70*	*47.75*	*39.85*

## Actual operations

The proposed hybrid method is suitable to run on an embedded system because of the low computation. There are no complex operations in the simplified PCNN model. We have developed a prototype system installed on a 210 in. warp-knitting machine. The system consists of six smart cameras and an HMI controller. The processing speed is about 5 frames per second, which can meet the real-time detection demands of warp-knitting machine. The actual operations proved the system is effective with a detection accuracy of 98.6 %.

## Conclusions

This paper presents an automatic fabric inspection system that consists of smart cameras and an HMI controller. The system can be applied to defect inspection for warp-knitting machine. The key part of the system is the hybrid inspection algorithm combining Gabor filters and PCNN with adaptive parameter setting. The performance of the system was verified on a warp knitting machine successfully. Future work will investigate the effectiveness of the proposed method for defect inspection of more complex fabrics.
